# Supplementary material for: Content-rich biological network constructed by mining PubMed abstracts
Source: BMC Bioinformatics. 2004 Oct 8;5:147. doi: 10.1186/1471-2105-5-147 (PMC528731; doi:10.1186/1471-2105-5-147)
Supplement: Additional File 2 — The original results of the above study (non-essential files are deleted to keep the file size under the limit set by BMC bioinformatics). [file 1471-2105-5-147-S2.bz2 › chilibotAdditionalFile2/dip20/448ID9115216E10455/html/CCR1_CCL2.html]

 


 **CCR1** and **CCL2** 
  
Found 155 abstracts in PubMed, retrieved 20.  
 

 What does Google say? 
 PDF only 
| .edu only 

---

**Interactive relationship** (e.g. stimulation, inhibition, etc)

**Neutral relationship**- Functional studies and binding experiments using biotinylated monocyte chemoattractant protein MCP 1  [ **CCL2** ]   **CCL2**  indicate that  **CCL2**  could be a candidate chemokine ligand for glial L CCR  [ **CCR1** ] .  Ref: 12555200 Glia, 2003
- Expression of L CCR  [ **CCR1** ]  in HEK 293 cells reveals functional responses to  **CCL2** , CCL5, CCL7, and CCL8.  Ref: 12885941 J Leukoc Biol, 2003
- Biotinylated  **CCL2**  binds to L CCR  [ **CCR1** ]  expressing cells, and transfection experiments with an L CCR  [ **CCR1** ]  green fluorescent protein fusion protein showed L CCR  [ **CCR1** ]  expression in the membranes of recombinant HEK 293 cells.  Ref: 12885941 J Leukoc Biol, 2003
- The G protein coupled receptor CCR  [ **CCR1** ]  2B is probably the most important MCP 1  [ **CCL2** ]  receptor in vivo, and loss of MCP 1  [ **CCL2** ]  effector function alone is sufficient to impair monocytic trafficking in inflammation models.  Ref: 12556201 Expert Opin Ther Targets, 2003
- The present findings suggest that MCP 1  [ **CCL2** ] , RANTES, and IP 10 may participate in the pathogenesis of mercuric chloride induced tubulointerstitial fibrosis with mononuclear cell infiltration, via CCR2,  **CCR1**  or CCR5, and CXCR3, respectively.  Ref: 12834626 Exp Mol Pathol, 2003

**Non-interactive relationship** (e.g. studied together, co-existance, homology, etc.)

- CD14 DCs retain unique features of tissue DCs, as evidenced by the gene expression profile of no CCR7 but more  **CCR1**  and no TARC but abundant MCP1  [ **CCL2** ]  and Eta 1.  Ref: 12176896 Blood, 2002
- We used radioligand binding and 35S GTPgammaS exchange assays in membranes from a cell line transfected to express  **CCR1**  Ba F3 hCCR1 to characterize a panel of chemokines HCC 1, MIP 1alpha, MIP 1beta, MIP 1delta, MPIF 1, MCP  [ **CCL2** ]  2, MCP  [ **CCL2** ]  3, and RANTES as  **CCR1**  ligands.  Ref: 12381680 Br J Pharmacol, 2002
- Only CpG ODN exacerbated HAF GN with an increase of glomerular macrophages, which was associated with massive albuminuria and increased renal MCP 1  [ **CCL2** ]   **CCL2** , RANTES CCL5,  **CCR1** , CCR2, and CCR5 mRNA expression.  Ref: 12538732 J Am Soc Nephrol, 2003
- In parallel,  **CCR1**  and CCR5 expression is down regulated and CXCL8,  **CCL2** , CCL3 and CCL5 production is up regulated.  Ref: 12548563 Eur J Immunol, 2003
- Synthetic MCP  [ **CCL2** ]  analogs corresponding to the MMP cleaved forms bound CC chemokine receptor CCR  [ **CCR1** ]  2 and CCR  [ **CCR1** ]  3, but lacked chemoattractant activity in pre B cells transfected with CCR  [ **CCR1** ]  2 and CCR  [ **CCR1** ]  3 or in THP 1 monocytic cells, a transformed leukemic cell line.  Ref: 12149192 Blood, 2002
- Several selective small molecular weight CCR  [ **CCR1** ]  2B antagonists and MCP 1  [ **CCL2** ]  blocking antibodies have been described.  Ref: 12556201 Expert Opin Ther Targets, 2003
- The mRNAs for MIP 1alpha and MCP 1  [ **CCL2** ] , as well as their cognate receptors  **CCR1**  and CCR2, respectively, were localized in resident and infiltrating macrophages in numbers commensurate with those of F4 80 immunopositive macrophages in adjacent tissue sections.  Ref: 12444600 J Neurosci Res, 2002
- Paraffin embedded specimens of 17 resected cardiac myxomas were immunohistochemically stained for MCP 1  [ **CCL2** ] , CC chemokine receptor 2 CCR  [ **CCR1** ]  2, TP, CD31, and CD68.  Ref: 12520153 Circ J, 2003
- We studied polymorphisms in the genes for monocyte chemoattractant protein 1  [ **CCL2** ]  MCP 1  [ **CCL2** ]  and CC chemokine receptor CCR  [ **CCR1** ]  2 in 171 Parkinson s disease PD patients and 340 controls.  Ref: 12889089 Mov Disord, 2003
- In the tumor stroma, the major source of MCP 1  [ **CCL2** ] , TP and CCR  [ **CCR1** ]  2 was macrophages, and the sites of positive staining for MCP 1  [ **CCL2** ] , TP and CCR  [ **CCR1** ]  2 matched in most of the myxomas.  Ref: 12520153 Circ J, 2003
- In J774 cells, CpG ODN induced the chemokines MCP 1  [ **CCL2** ]   **CCL2**  and RANTES CCL5 and the chemokine receptors  **CCR1**  and CCR5.  Ref: 12538732 J Am Soc Nephrol, 2003
